# HEALTH AND ENERGY OUTLOOK PREDICT FUNCTIONAL HEALTH 20 YEARS LATER

**DOI:** 10.1093/geroni/igae098.3273

**Published:** 2024-12-31

**Authors:** Morgan Taylor, Margie Lachman

**Affiliations:** Brandeis University, Waltham, Massachusetts, United States; Brandeis University, Waltham, Massachusetts, United States

## Abstract

Research has shown that older adults who hold positive self-perceptions of aging have better cognitive and health outcomes. However, few studies have investigated this in a long-term longitudinal and full adult lifespan sample. We used data from the Midlife in the United States (MIDUS) study to investigate whether outlooks of one’s health and energy assessed at MIDUS 1 (M1; 1995-1996) would be associated with health limitations (e.g., trouble carrying groceries, difficulty climbing stairs) decades later at MIDUS 3 (M3; 2013-2014). At M1, participants were asked to rate their health and energy on a scale from 0 to 10 at three different time points: the past (10 years prior), the present, and the future (10 years hence). To determine their outlook, we computed a difference score by subtracting participants’ past rating from their future rating. Scores ranged from -10 to 10 and represented a 20 year change that modeled the actual gap between M1 and M3. Using multiple linear regression, we found that health and energy outlook was negatively associated with residual change in health limitations at M3, such that those with more positive outlooks reported fewer health limitations, controlling for age, sex, education, and baseline health limitations. Participant age did not interact with this effect, demonstrating that positive outlooks can benefit the health of individuals across the adult lifespan.

